# The microbiological quality of air improves when using air conditioning systems in cars

**DOI:** 10.1186/1471-2334-10-146

**Published:** 2010-06-01

**Authors:** Ralf-Peter Vonberg, Petra Gastmeier, Björn Kenneweg, Hinrich Holdack-Janssen, Dorit Sohr, Iris F Chaberny

**Affiliations:** 1 Institute for Medical Microbiology and Hospital Epidemiology, Hannover Medical School, Hannover, Germany; 2 Institute for Hygiene and Environmental Medicine, Charité - University Medicine Berlin, Berlin, Germany; 3 Institute of Automotive Engineering, University of Applied Sciences, Braunschweig and Wolfenbüttel, Germany

## Abstract

**Background:**

Because of better comfort, air conditioning systems are a common feature in automobiles these days. However, its impact on the number of particles and microorganisms inside the vehicle - and by this its impact on the risk of an allergic reaction - is yet unknown.

**Methods:**

Over a time period of 30 months, the quality of air was investigated in three different types of cars (VW Passat, VW Polo FSI, Seat Alhambra) that were all equipped with a automatic air conditioning system. Operation modes using fresh air from outside the car as well as circulating air from inside the car were examined. The total number of microorganisms and the number of mold spores were measured by impaction in a high flow air sampler. Particles of 0.5 to 5.0 μm diameter were counted by a laser particle counter device.

**Results:**

Overall 32 occasions of sampling were performed. The concentration of microorganisms outside the cars was always higher than it was inside the cars. Few minutes after starting the air conditioning system the total number of microorganisms was reduced by 81.7%, the number of mold spores was reduced by 83.3%, and the number of particles was reduced by 87.8%. There were no significant differences neither between the types of cars nor between the types of operation mode of the air conditioning system (fresh air vs. circulating air). All parameters that were looked for in this study improved during utilization of the car's air conditioning system.

**Conclusions:**

We believe that the risk of an allergic reaction will be reduced during use also. Nevertheless, we recommend regular maintenance of the system and replacement of older filters after defined changing intervals.

## Background

Many cars are supplied with air conditioning (AC) systems these days. On the one hand AC systems increase the fuel consumption of the vehicle, but on the other hand AC systems provide several advantages for the driver. A moderate cooling by AC systems increases the comfort for all passengers especially at hot temperatures outside the car [1]. The level of the driver's concentration improves and the risk of an accident decreases [2]. Many AC systems contain filter systems for cleaning purpose. Usually, these filters consist of a combination of fleece (for the back draft of particles) and some layers of activated charcoal (to eliminate odours of other unwanted aerosolized components in the air from the outside). By this, the filter systems are meant to protect the passengers from pollution by industry, by traffic, and by various biological sources. Filters systems need to be checked and replaced regularly. Every two years at the latest, filters should be exchanged to prevent rupture and to limit growth of moulds and bacteria through the filtrating membranes [3]. The quality of filtration of filters in car's AC systems corresponds to the filter class F5 as it is usually used in air supply systems of buildings [4,5].

Compared to air supply systems in buildings, AC systems in vehicles come with some disadvantages due to the small space available in cars. Tiny air conduits and frequent changes of the direction of air flow in cars support deposition of airborne particles and microorganisms within the airways [6]. Once those particles or microorganisms happen to reach the cabin of the car, a possibility for allergic reactions (e.g., of the respiratory tract) for passengers exists in principle [7,8]. There are also some concerns that persons with some kind of a severe immunodeficiency may be at risk of an airborne infection caused by an AC system.

Aim of the present study was to determine the influence of AC systems in cars on the quality of filtered air from the outside and air re-circulating from the car's cabin under various conditions.

## Methods

### Time

01/2004 - 07/2006 (seasonal variations taken into account).

### Place

University of Applied Sciences Braunschweig/Wolfenbüttel (location Wolfsburg) and Hannover Medical School (regional variations taken into account).

### Vehicles

The cars are described in Table 1. All were equipped with a "Climatronic AC system" that automatically regulates temperature, quantity of ventilation, and distribution of air through the different air outlets. Air filters had been changed regularly according to the manufacturer's instructions.

**Table 1:** Cars used in this study

company name of car	type of car	type of fuel	year of construction	running distance on record (km)
Volkswagen Polo FSI	small car	gasoline	2003	10.000

Volkwagen Passat	station wagen	diesel	1998	110.000

Seat Alhambra	small van	gasoline	1997	175.000

### Pre-experiment under extreme humidity of the outside air

High humidity in the outside air (constantly 75% - 95% relative humidity) at a temperature of 30°C - 33°C was archived by placing a Volkswagen Polo FSI for 4 days into a closed container equipped with an air steam humidifier and a hygrograph. On the 4^th ^day, the number of fungal spores and particles in the cabin were determined before starting the AC system and during its use, as well as the bio burden in the surrounding air within the container.

### Pre-experiment using an old filter in the AC system

We used an additional Volkswagen Polo (for pre-experiments only), in which the air filter had not been changed regularly. We determined changes in microorganisms, the fungal spores, and particles during AC system use. After that the old filter got replaced by a new one and experiments were repeated.

### Use of AC systems in the main experiment

Regularly AC systems use air from outside the car. However, sometimes (e.g., if the outside air is heavily polluted) the use of re-circulating air from inside the cabin may be more appropriate. In order to evaluate the quality of air in both modes, the quality of air from the car's AC systems were tested under maximal work load using either fresh air from the outside (OA) and re-circulating air from the inside (IA). Furthermore, a variable (VAR) of the AC systems was simulated by switching first from OA to IA and then back to OA. Time intervals for testing the three modes (OA, IA, and VAR) are shown in Table 2. In addition to analyzing the air when leaving the AC system, we also took air samples from inside the cabin before starting the AC system so changes in the quality of air inside car as the AC system work could be measured. Air samples from outside the car were also taken to determine the bio burden of the air before entering the AC system.

**Table 2:** Plot of air sampling of the car's AC system inside the cabin when using either outside air (OA), inside air (IA), or variable use (VAR) of both types of air

time (min)	fresh air (outside) (OA)			re-circulating (inside) (IA)			variable (outside/inside) (VAR)			
	TSM	DG-18	particle	TSM	DG-18	particle	air	TSM	DG-18	particle
**no AC use**	●	●	●	●	●	●		●	●	●

**AC start**	●	●	●	●	●	●	OA	●	●	●
		
**1**	●			●				●	●	●
		
**2**	●	●	●	●		●				
		
**3**	●			●				●		●
		
**4**	●		●	●		●				
		
**5**	●	●		●	●			●	●	●
		
**6**	●		●	●		●				
		
**7**	●							●		●
		
**8**	●		●							
		
**9**	●							●		●
		
**10**	●	●	●							
		
**11**								●	●	●

**13**	●		●				IA	●	●	●
		
**15**								●		●
		
**16**	●		●							
		
**17**								●	●	●

**19**							OA	●	●	●
		
**20**	●	●	●							
		
**21**								●		●
		
**outside the cabin**	●	●	●	●	●	●		●	●	●

tryptic soy medium (TSM) indicator agar = evaluation of the total number of pathogens

dichloran glycerol (DG-18) agar = evaluation of the number of fungal spores

### Air sampling (microorganisms)

**Figure 1:**
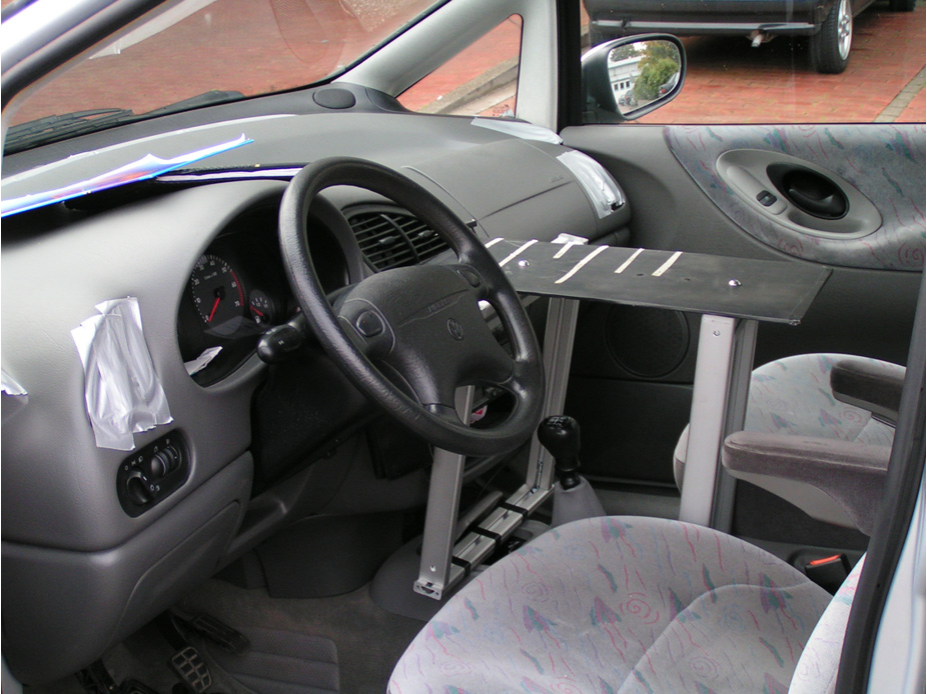
**Preparation of study vehicles**. Air outlets, that do not get sampled, are sealed by tape. An adjustable board is placed between the front seats.

Figure 1 shows the preparation of cars. All air outlets (including the front wind shield and leg spaces of front seats) of the vehicle other than the single one sampled (between the front seats) were closely taped before the experiment in order not to miss air from the AC system. Two RCS High Flow Air Samplers (Biotest, Dreieich, Germany) were used simultaneously. Via impaction the total number of pathogens (tryptic soy medium (TSM) indicator agar, Biotest) and the number of fungal spores (dichloran glycerol (DG)-18-indicator agar, Biotest) were measured. Sampling devices were placed on a self-made board that could be adjusted in height and width for the different types of cars (Figure 1). AC systems were always run at maximal output. The distance between air outlets and samplers was 15 cm. Sampling took 1 minute min each with a volume of 50 L.

### Air sampling (particles)

Airborne particles (between 0.5 and 5.0 μm in diameter) were measured by a laser particle counter Met One 3313 (MP-Messtechnik GmbH, Adelzhausen, Germany). The tube of the counter was also positioned on the variable board.

### Identification of microorganisms in the laboratory

Differentiation of microorganisms was performed in the Institute for Medical Microbiology and Hospital Epidemiology (Hannover Medical School) according to standard operation protocol as certified by the German DIN EN ISO 15189. TSM indicator agar stripes for the overall concentration of microorganisms were incubated for 48 hours at 36°C and colony-forming units (CFU) were counted. TSM indicator agar stripes were then incubated for additional 5 days at room temperature to check for slow-growing moulds. DG-18-indicator agar stripes for the detection of fungi were incubated for 7 days at room temperature and CFU of mould were counted.

### Statistical analysis

To measure the effect of the AC system on the total number of microorganisms and the number of mould's spores, the logarithmic quotients of the number before starting the AC system divided by the number after using the AC system in all sequences of air sampling are calculated resulting in an approximately normal distributed variable. The quotient of the number of airborne particles before starting the AC system divided by the number of airborne particles after using the AC system can also be assumed as approximately normal distributed. We performed analysis of variance to prove the influence of the type of car or the operation mode and calculated Gabriel's confidence intervals for the logarithmic quotients or the quotients itself as parameters of interest respectively. For the total number of microorganisms and the number of mould's spores the antilogarithm of means and confidence intervals lead to geometric means and confidence intervals for the quotients as parameters of interest. Further calculations resulted in characteristics of a proportional decrease of the numbers of interest with corresponding confidence intervals.

## Results

The first pre-experiment showed that an extreme humidity leads to an increase of fungal spores inside the cabin (1,400 CFU per m^3^) before the AC system was started compared to 540 CFU per m^3 ^fungal spores in the surrounding air in the container. However, when starting the AC system the number of spores immediately dropped to 1,000 CFU per m^3 ^and quickly reached low levels of fungi thereafter (380 CFU per m^3 ^after 3 minutes and 60 CFU per m^3 ^after 21 minutes of use). The number or particles was also increased in the cabin initially (before staring the AC system: 23,192 particles per m^3 ^inside the car vs. 9.566 particles per m^3 ^in the surrounding air). However, once again the values dropped quickly after the AC system was switched on (down to 1.553 particles per m^3 ^after 21 minutes of use).

The second pre-experiment showed that the number of microorganisms inside the car increased by 67% when using the old filter (120 CFU before to 200 CFU per m^3 ^after use of the AC system), the number of fungal spores even doubled during use of the AC system (from 10 to 20 CFU per m^3^), the number of particles decreased at the same time by 57% (from 18,109 to 7,872 per m^3^). The quality of air improved when the old filter was replaced by a new air filter. The number of microorganisms now decreased by 57% (from 140 to 60 CFU per m^3^), fungal spores got completely eliminated (from 20 to 0 CFU per m^3^), and the number of particles decreased by 80% (from 27.181 to 5.401 per m^3^).

An overall number of 32 sequences of air sampling were performed in the main experiment (8 in the Volkswagen Passat, 14 in the Volkswagen Polo FIS, and 10 in the Seat Alhambra) using regularly changed filter systems only. The concentration of airborne microorganisms was higher in the air outside the cabin compared to inside the cabin at all times.

**Figure 2:**
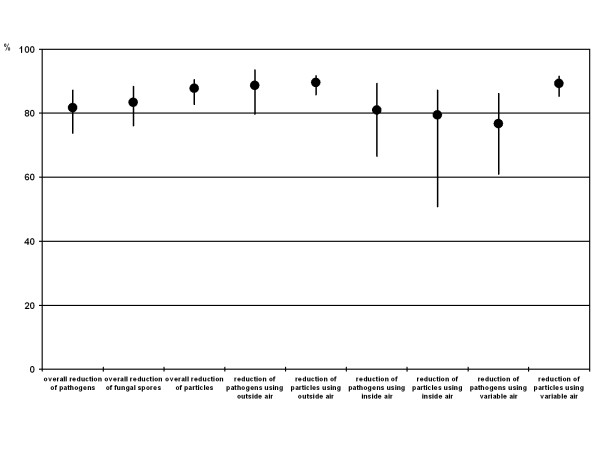
**Change in the concentration of particles and microorganisms**. Geometric mean (for pathogens and fungal spores)/arithmetic mean (for particles) and 95% confidence interval of the reduction of total number of pathogens, number of fungal spores, and number of particles during use of different modes of the AC system (outside air, inside air, variable use).

In all sequences of air sampling the total number or microorganisms decreased (mean reduction: 81.7%), the number of mould's spores decreased (mean reduction: 83.3%), and the number of airborne particles decreased (mean reduction: 87.8%) within few minutes after starting the AC system. We did not detect *Aspergillus *spp. in any of our samples. Changes in the quality of air in the cabin during the use of the car's AC system are shown in Table 3 and Figure 2. When fresh air from outside the car was used, the reduction of microorganisms and particles was slightly (though not significantly) higher compared to the use of re-circulating air.

**Table 3:** Mean reduction of microorganisms under various conditions of the car's AC system

	source of air used in the AC system			
		
	outside	inside	variable	overall reduction
pathogens	88,6%	81,0%	76,7%	81,7%

particles	89,5%	79,5%	89,3	87,8%

We did not find differences between the various types of cars in our study. In the Volkswagen Passat the mean reduction of microorganisms and particles were 80.5% (CI95%: 63.2 - 89.6) and 87.8% (81.4 - 91.0) respectively. In the Volkswagen Polo FIS the decrease of microorganisms was 81.3% (69.9 - 88.4) and the particles got diminished by 88.7% (84.4 - 91.2). Improvement of the quality of air in the Seat Alhambra was 85.3% for microorganisms (74.1 - 91.7) and 86.6% for particles (79.9 - 89.9).

## Discussion

Meanwhile AC systems are present in all kinds of cars. The advantages of AC system such as comfort by cool air in the summer time are well-known. However, the potential risk of contamination of the air in the cabin by microorganisms by the AC system has yet rarely been examined.

The usage intervals of filters that are used in the air conduits of the AC system as well as the frequency of use of the AC system have a major impact on the quality of air. In an experiment mould spores were placed on a polycarbonate-micro fibre filter for cars; when using dynamic conditions (air flow through the filter) no growth of moulds was detected, but under static conditions (no air flow through the filter) there was growth of mycelia [9]. Fungicide layers on filter membranes may reduce growth of moulds [10], but other components of the AC system such as the evaporator or sealing material may also serve as matrix for microbiological growth e.g., for moulds [11]. Once biofilm formation has taken place in the AC system, volatile organic compounds derived from microorganisms may enter the cabin of the vehicle through the airways [12], and represent an potential threat for predisposed passengers [13].

The present study shows an enormous improvement of the microbiological quality of air when using the AC system in all cars tested. Thus, our data confirm the findings of Kumar et al. [14], who also determined a significant reduction of mould spores in a car's cabin when using the AC system. Muilenberg et al. report a decrease of the concentration of airborne particles around the front seats when using an AC system [15].

One limitation of our study is that we cannot evaluate whether the air of the AC systems contained antigenic components of microorganisms, since we only checked for growth of pathogens. That is why we recommend examination of the AC system as soon as any suspicious kind of odour gets recognized during use (it might be necessary that air filters and/or other components of the AC system need to become exchanged). In the presented study we never noticed any unusual smell derived from the AC system.

Nevertheless, may we remind that the cabin itself can also represent a major source of volatile organic compounds [16], but a well-functioning AC system should be able to reduce those substances (by dilution) for the benefit of the passengers [17].

## Conclusions

If examined and exchanged regularly, AC systems in cars may significantly improve the quality of air inside the car's cabin. By this they seem suitable to reduce the subsequent risk of allergic diseases of passengers.

## Competing interests

This research project was promoted by the federal government organization AGIP (Arbeitsgemeinschaft Innovative Projekte der angewandten Hochschulforschung beim Ministerium für Wissenschaft und Kultur des Landes Niedersachsen) between June 2004 and Mai 2006 with a total support of EUR 115,000. Partner from industry side was Otto Egelhof GmbH, Stuttgarter Str. 60, 70736 Fellbach, Germany.

## Authors' contributions

RV participated in the interpretation of the data and helped to draft the manuscript. PG, HHJ, and IFC participated in the design of the study. BK participated in the acquisition of the data. DS performed statistical analysis. All authors read and approved the final manuscript.

## Pre-publication history

The pre-publication history for this paper can be accessed here:

http://www.biomedcentral.com/1471-2334/10/146/prepub
